# Nanoscale Probing of the Organic Binder in Artists’ Paint Layers: Organic Phases and Chemical Heterogeneity

**DOI:** 10.1021/acsami.4c16430

**Published:** 2025-01-24

**Authors:** Rafaella Georgiou, Alexandre Dazzi, Jeremie Mathurin, Celia Duce, Patrick Dietemann, Mathieu Thoury, Ilaria Bonaduce

**Affiliations:** † Department of Chemistry and Industrial Chemistry, Università di Pisa, via Moruzzi 13, 56124 Pisa, Italy; ‡ Department of Physics, University of Wisconsin-Madison, 1150 University Avenue, 53706 Madison, Wisconsin, United States; ¶ Institut de Chimie Physique, UMR8000, Université Paris-Saclay, CNRS, 91405 Orsay, France; § Bayerische Staatsgemäldesammlungen, Doerner Institut, Richard-Wagner-Str. 1, 80333 Munich, Germany; ∥ CNRS, ministère de la Culture, UVSQ, MNHN, UAR3461, Université Paris-Saclay, Institut photonique d’analyse non-destructive européen des matériaux anciens, 91192 Saint-Aubin, France

**Keywords:** mixed-media paints, proteins, drying oil, oil paint, *tempera*, *tempera grassa*, Renaissance artworks, nanoinfrared microscopy

## Abstract

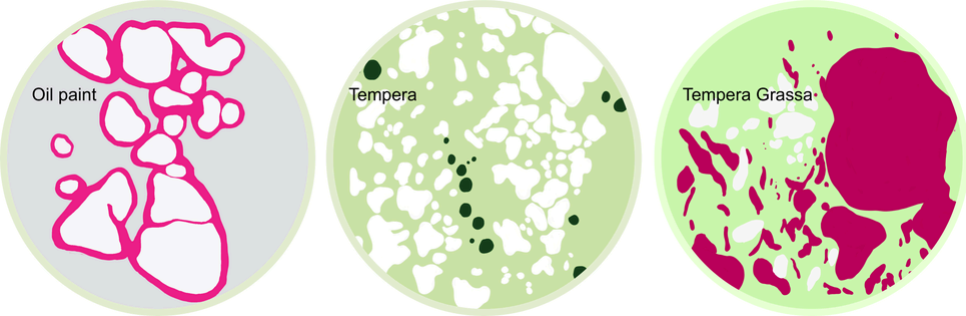

Understanding paint structures at the nanoscopic level can address key questions related to artistic techniques, paint formulation, and long-term preservation of artworks. This involves examining spatial chemical complexity, the formation of molecular networks, and interactions between organic and inorganic constituents. Depending on the paint preparation methods, proteins and drying oils, the most common binders in traditional artistic practices, can be integrated to produce paints with diverse structures and nanoscale chemical intricacies. In this study, we utilize atomic force microscopy-based infrared spectroscopy (AFM-IR) to investigate the spatial chemical complexity and reaction pathways of organic species in artists’ paints, including oil, *tempera*, and mixed-media *tempera grassa*. By analyzing these paints at the nanoscale, we established connections between their structural organization, chemistry, and formulation.

## Introduction

Decoding the chemical nature of organic components within artists’ paints is essential for tackling a series of questions critical to the comprehension and preservation of our tangible heritage. Although nanoscale speciation, elemental characterization, phase identification, and mapping of inorganic components have yielded essential insights in heritage studies,^1^ understanding of organic components at that scale remains under-researched. Probing the organic chemistry of artists’ paints at the nanoscale allows us to understand their chemical complexities, including multiscale spatial variations in chemical composition (i.e., multiscale heterogeneity), variations in the relative abundance of individual components (both organic and inorganic), and the intricate chemical composition of diverse chemical groups within the examined volume of paints.

Within the framework of organic materials utilized in Italy in the 15th century, grasping the simultaneous or combined use of proteins and drying oils is crucial to deciphering the production modes of oil, *tempera*, and mixed-media paints (e.g., *tempera grassa*) used to craft masterpieces of that era. Renowned artists of the time, such as Sandro Botticelli (1445–1510), Domenico Ghirlandaio (1449–1494), Leonardo da Vinci (1452–1519), Giovanni Bellini (1430–1516), and Antonello da Messina (1430–1479), incorporated the oil medium into their daily workshop practice and continued to use eggs in parallel or in combination with oil to produce paints of great complexity.^2−4^

The selection of binder is intimately connected with the artist’s painting technique. Egg and oil have different chemical compositions and properties that affect the formation of the paint. Egg *tempera*, a mixture of whole egg, yolk, or egg white with pigments, dries fast and does not allow for any blending of colors; thus, the paint was usually applied in characteristic hatched strokes. On the other hand, oil paint, a mixture of a drying oil with pigments, takes longer to dry and allows blending and further manipulation of the paint hours, even days after its application to the support.

Egg and oil may be combined in various ways.^5−7^ For example, a *tempera grassa* is an emulsion that can be created by gradually adding oil to a *tempera* paint.^5,6^ In fresh paint, when using egg yolk and raw linseed oil, oil droplets belong to the disperse phase, and aqueous egg yolk is the continuous phase.^5,6^ Alternatively, egg can be added to oil paints, either as a coating for pigment particles prior to dispersion in the oil phase or as capillary bridges between particles after the oil paint has been prepared.^7^ The different formulations result in paints with immensely diverse rheological and chemical properties, which can be attributed to the micrometric and nanometric structures obtained through various formulations and preparation methods.^6,7^

Deciphering the molecular networks formed, localizing organic and inorganic species in situ, and understanding the interaction between organic and inorganic components, as well as among diverse organic constituents, offer valuable insights into artistic techniques, paint formulations, and preservation strategies. Chromatographic mass spectrometric techniques allow the molecular characterization of complex organic samples with high sensitivity and specificity,^8−10^ yet, they do not address spatial chemical complexity. Recent progress in scientific analysis, particularly in chemical imaging methods, can be useful to reassess the development of materiality and painting techniques in 15th-century Italian painting. Numerous techniques, such as Fourier transform infrared spectroscopy (FT-IR), Raman spectroscopy, nuclear magnetic resonance (NMR), and time-of-flight secondary ion mass spectrometry (ToF-SIMS) are widely employed for determining the microstructure of complex organic materials via point analysis and chemical imaging.^11−15^ Yet, each technique has experimental constraints that include the depth, sensitivity, and spatial resolution probed.

Infrared microspectroscopy, used to analyze the chemical composition of various layers of paint in aged samples, is restricted by diffraction-limited resolution, with the smallest achievable pixel size typically exceeding 2.5 μm.^16−18^ AFM-IR is an emerging technique that combines the chemical sensitivity and specificity of infrared spectroscopy (IR) with the spatial resolution of atomic force microscopy (AFM), achieving resolutions below 20 nm, which is more than 2 orders of magnitude below the diffraction limit.^19^ The microstructures within diverse paint systems are anticipated to be smaller than the achievable spatial resolution of FTIR. The AFM-IR imaging achieves the nanoscale spatial resolution necessary for studying the chemical heterogeneity of organic binders in paint layers, with the resolution being limited only by the radius of the AFM probe tip. In this technique, an IR laser pulse reaches the sample surface and is absorbed, leading to localized thermal expansion detected with the AFM probe.^19^

Up to now, few studies have investigated paint samples at the nanoscale. Morsch et al. employed AFM-IR to map (with a pixel size of ≈30 nm) and differentiate UV-induced degradation of two grades of titanium dioxide (i.e., rutile and anatase TiO_2_) model linseed oil paints.^20^ Ma et al. investigated the formation and aggregation of soap in a 23-year-old naturally aged zinc-containing soft titanium white oil paint (P250) using AFM-IR in tapping mode with a spatial resolution of ≈10 nm.^21^ Optical photothermal induced resonance (OPTIR) and AFM-IR with spatial resolutions of ≈500 nm and ≈10 nm, respectively, were combined to study a sample collected from a 19th-century painting by Corot,^22^ for the identification and localization of chemical species in the top layer of the artwork.

Here, we employ AFM-IR to elucidate the chemistry, molecular structures, and spatial distribution of organic constituents (i.e., proteins, oils) in oil, *tempera*, and *tempera grassa* paints with a nanoscale spatial resolution that is proven critical for tackling their structure and chemistry. We visualize (i) the preferential accumulation of polar groups near pigment particles in a lead white oil paint layer; (ii) the intricate structure of natural nanoassemblies and microassemblies retained in the egg yolk lead white *tempera* paint layer, and (iii) the heterogeneous chemistry at the pigment–oil and oil–protein interfaces in a *tempera grassa* paint layer.

## Materials and Methods

### Materials and Paint Preparation

The paints were prepared by Ophélie Ranquet using lead white (Kremer Pigmente, Germany) and synthetic ultramarine blue (Blu Oltramare Puro M, Abralux Colori Beghè, Italy) as pigments. For oil paints, pigments were mixed with cold pressed linseed oil (Maimeri, Italy) and ground with a flat muller on a glass plate, starting as a stiff paste and gradually achieving a smooth consistency. Tempera paints were made by combining lead white with fresh egg yolk and distilled water and grinding the mixture to a well-dispersed paint with intermittent addition of water to compensate for quick drying. *Tempera grassa* was prepared by enriching freshly made tempera paint with drops of linseed oil while grinding for uniformity. The prepared paints were applied to the glass slides as thin, even layers using a palette knife and allowed to dry under ambient conditions. Detailed compositions and aging of the paint layers are provided in Supporting Information Table S1. The microsamples studied were collected from each of the aged paint layers and embedded in epoxy resin using EpoFix hardener (AGB8790H) and EpoFix resin (AGB8790R) supplied by Agar Scientific (United Kingdom). The embedded samples were subjected to microtoming, exposing the stratigraphy of the samples with minimal surface roughness.

### Challenges Due to the Sample Preparation

In this work, we use an epoxy resin (i.e., EpoFix resin (Agar Scientific, U.K.)) to embed the paint samples. The stratigraphy of the sample is then exposed by microtoming the block surface to achieve a flat surface (see Supporting Information Figures S1, S2, S3, and S4). However, the commonly used cross-section preparation method, widely applied to historical painting samples for easier handling, introduces certain limitations. During the embedding and microtoming steps, the resin can penetrate the porous paint layers and smear across the surface of the section, especially if it is not fully cured. This contamination complicates the characterization of the organic components.

In this AFM-IR study, which employs the tapping mode, the probing depth is limited to the first few hundred nanometers. Recent research by Dazzi et al. indicates that, in tapping mode, the probe depth for poly(methyl methacrylate) (PMMA) is around 25 nm, while contact mode can extend this depth to several micrometers.^23^ Although contact mode would have been advantageous for mitigating the effects of surface contamination from the embedding medium, we opted against it because of the sample’s diverse mechanical properties, with both organic and inorganic phases present, which would pose challenges during acquisition.

The characterization of the organic components of the samples is complicated as a result of the overlap in the absorption bands between the embedding resin and paint binders. The spectrum of epoxy resin can be found in Supporting Information Figure S5. It shows strong absorption bands at 1097 cm^–1^ and 1677 cm^–1^ and a medium absorption band at 1161 cm^–1^. Thus, in the interpretation of the samples’ spectra, we mainly focus on nonoverlapping bands or underline the presence of overlapping bands when discussed. In future analyses, whenever possible, alternative methods for handling the sample during preparation should be explored. For example, the use of sulfur embedding combined with ultramicrotomy, used in other AFM-IR studies, could be considered to avoid synthetic resin contamination.^24^

### Atomic Force Microscopy-Based Infrared Spectroscopy

AFM-IR measurements were conducted at the Institut de Chimie Physique (Université Paris-Saclay) using a Bruker IconIR, integrating a tunable mid-IR laser (900–1900 cm^–1^) with an atomic force microscope. The IR laser induces localized heating expansion upon absorption by the sample, generating a mechanical force on the AFM probe proportional to the absorption coefficient, which is used to produce the IR spectra. Tapping mode scanning, using a Nanosensors cantilever (PPP-NCHAu-MB, 50 N/m, 270 kHz), allowed the simultaneous collection of topographic images and IR maps at fixed laser wavenumbers. A PLL system compensated for frequency shifts caused by tip–sample interactions, maintaining constant oscillation amplitude and enabling accurate IR mapping. The phase shifts recorded by PLL revealed local mechanical property variations. AFM-IR spectra were collected by holding the tip stationary and tuning the IR laser across wavenumbers with a resolution of 1 cm^–1^. Additional details are provided in the Supporting Information.

## Results and Discussion

### Oil Paint

An artist’s oil paint is made by grinding the pigment with a drying oil (e.g., linseed oil). Linseed oil, often used as a binder in artists’ paints, is a plant oil consisting of triglycerides containing primarily polyunsaturated fatty acids. Linseed oil hardens over several weeks, as components of the oil polymerize to form an insoluble matrix. In this work, model oil paint layers are prepared with lead white or synthetic ultramarine blue pigments and cold pressed linseed oil (Supporting Information Table S1, Figure S1).

**Figure 1:**
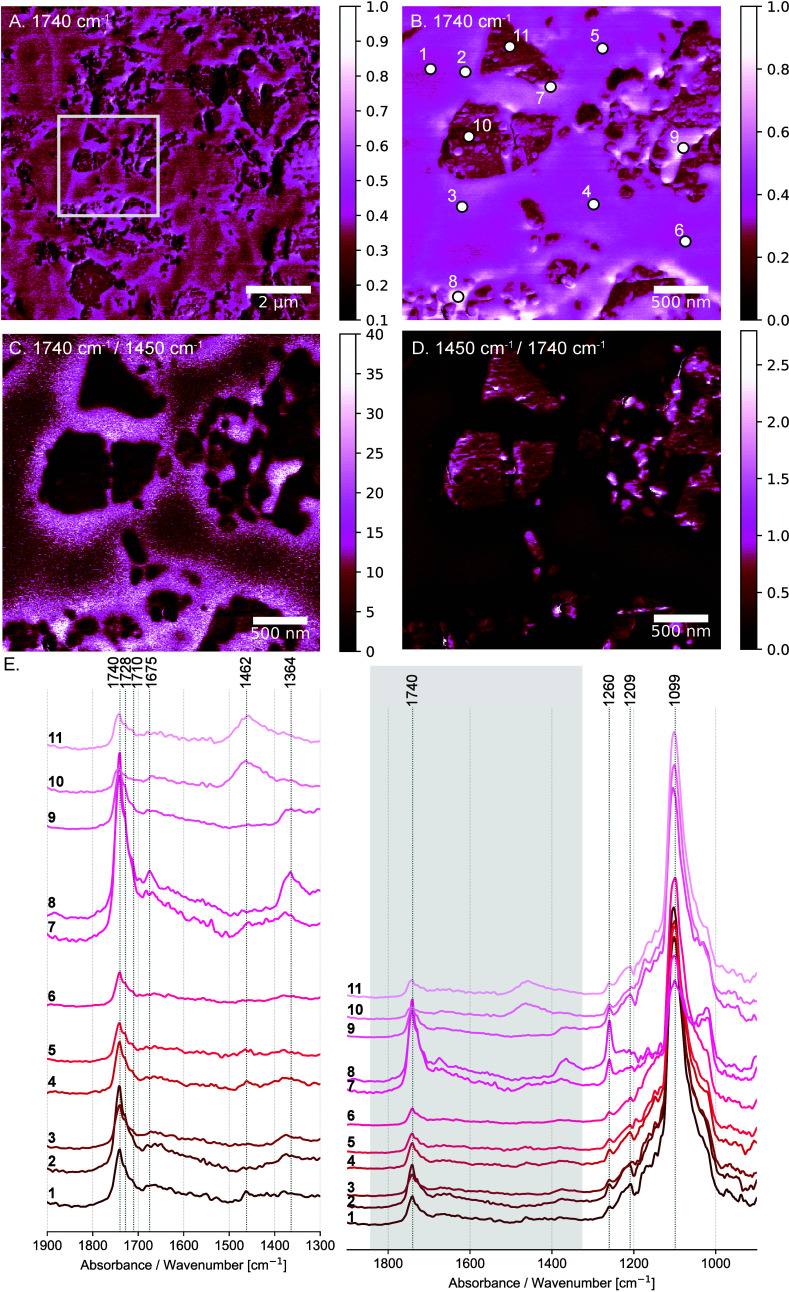
Nanoresolved structure of the lead white oil paint layer. (A) The AFM-IR absorption image at 1740 cm^–1^ illustrates the spatial arrangement of ester groups (C=O stretching vibration) within the oil binder. (B) Higher magnification AFM-IR absorption image at 1740 cm^–1^ (C=O stretching vibration). (C) The 1740 cm^–1^/1450 cm^–1^ ratio image illustrates the preferential accumulation of the polar groups (e.g., esters) near the pigment particles. (D) The 1450 cm^–1^/1740 cm^–1^ ratio image demonstrates that the aliphatic groups (CH_2_ scissoring vibrations and/or CH_3_ asymmetric deformations at 1450 cm^–1^) are concentrated away from the pigment particles, showing a directional gradient in their spatial organization. (E) The AFM-IR spectra at spots 1–11, as identified in (B), show increased 1740 cm^–1^ signals near pigment particles (spots 7 and 8). Shoulders at 1728 cm^–1^ and 1710 cm^–1^ suggest additional carbonyl groups (e.g., carboxyls, ketones, aldehydes) within the oil binder. Spectra are partially obscured by the epoxy embedding medium interference, particularly in the region of 1200–1000 cm^–1^ (Supporting Information Figure S5).

The AFM-IR nanoscale images emphasize the impact of the lead white pigment particles in the oil paint chemistry (Figures 1, S6, S7, and S8). Lead compounds are known to influence the drying behavior and physical properties of oil paints. Lead white has a catalytic effect in the reactivity of polyunsaturated triglyceride fatty acids; it accelerates oxygen uptake and autoxidation in an oil paint.^25,26^ What becomes apparent at this scale is the increased absorption intensity of various carbonyl groups surrounding the lead white pigment particles (Figure 1A,B,C, Supporting Information Figure S7). The absorption band at 1740 cm^–1^ is attributed to the C=O stretching vibration of the ester groups in the triglycerides (Figure 1A,B). The shoulders at 1728 cm^–1^ and 1710 cm^–1^ are attributed to different carbonyl groups, such as aldehydes, ketones, and carboxylic acids, which are formed and increase with curing and hydrolysis^27,28^ (Figure 1F, Supporting Information Figure S7).

The 1740 cm^–1^/1450 cm^–1^ ratio image illustrates the preferential distribution of functional groups within the paint binder. Polar groups, primarily esters along with aldehydes, ketones, and carboxylic acids (C=O stretching vibrations at 1740 cm^–1^ with shoulders at 1728 cm^–1^ and 1710 cm^–1^), accumulate near the pigment particles, indicating a strong affinity (Figure 1C, Supporting Information Figure S7). Conversely, the aliphatic groups (CH_2_ scissor vibrations and/or CH_3_ asymmetric deformations at 1462 cm^–1^) are distributed away from the pigment particles, suggesting a directional gradient in their spatial organization (Figure 1D). We initially collected the AFM-IR absorption map at 1450 cm^–1^, anticipating a peak indicative of aliphatic groups and lead white particles. Subsequently, we collected spectra specifically from the regions of interest at this wavelength, which showed a precise contribution at 1462 cm^–1^. The 1450 cm^–1^ image, although not at the peak maximum, shows the distribution of the C=O stretching of the inorganic carbonate in lead white in addition to the CH_2_ scissor vibration and/or the CH_3_ asymmetric deformation in the oil binder (Figure 1D, Supporting Information Figure S6C).

**Figure 2:**
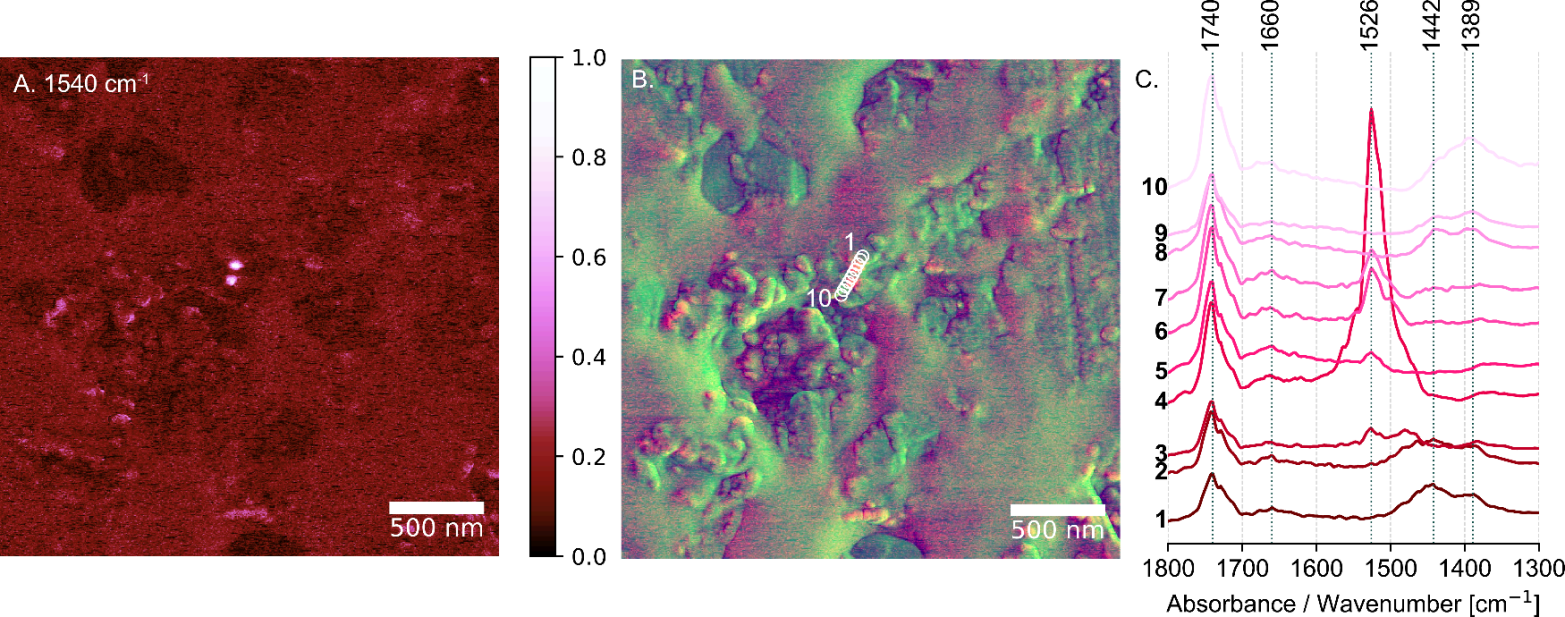
Formation of lead carboxylates in the lead white oil paint layer. (A) The hotspots observed in the 1540 cm^–1^ AFM-IR absorption image indicate the presence of lead carboxylates within the oil binder. (B) The overlay of the phase signal image (green) and the AFM-IR absorption image at 1540 cm^–1^ (red) illustrates the formation of nanometric-sized lead soaps near the surface of pigment particles. (C) The AFM-IR spectra collected at spots 1–10, as identified in (B), exhibit an additional absorption signal at 1526 cm^–1^ (spots 4, 6, 7), attributed to the asymmetric COO^–^ stretching in crystalline lead carboxylates.

The AFM-IR absorption images shown in Figure 1A,B,C illustrate a preferential distribution of the oil polar groups around the pigment particles. The lead white dissolves in the drying oil to form lead soaps, and we may expect that coordination of lead ions by carboxyl moieties precedes the saponification step. In fact, it is also possible to detect the nucleation sites of metal carboxylates (see Figure 2, Supporting Information Figure S8). The absorption at 1526 cm^–1^ (Figure 2C, spectra 4, 6, 7) corresponds to the asymmetric COO^–^ stretching in crystalline lead soaps.^29^

In Figure 2, we include the absorption map available at 1540 cm^–1^ which, although slightly offset from the peak maximum, still provides evidence of lead soap formation. The frequency of the asymmetric carboxylate stretch vibration band varies due to the evolution of the carboxylate structure during the transition from the amorphous state to the crystalline state.^29^ We initially collected the AFM-IR absorption map at 1540 cm^–1^, anticipating a peak indicative of the formation of a lead soap. Subsequently, we collected spectra specifically from the regions of interest at this wavelength, which confirmed a precise contribution at 1526 cm^–1^. This approach helped us verify the presence and exact location of the absorption feature. The overlay of the phase signal image (green) and the AFM-IR absorption image at 1540 cm^–1^ (red) shows the emergence of nanometric-sized lead carboxylates (approximately 60 nm), which are distributed close to the interface between the pigment particles and the binder (see Figure 2A,B, Supporting Information Figure S8).

Synthetic ultramarine blue, a sulfur-containing sodium aluminum silicate ((NaCa)_8_[Al_6_Si_6_O_24_](SO_4_,S,Cl)_2_), when mixed with a drying oil, slows the drying process and is not prone to the formation of metal soaps. In the synthetic ultramarine blue oil paint under study, the various carbonyl groups are distributed quite homogeneously in the organic phase (see Supporting Information Figures S9 and S10).

### Egg Tempera

Egg *tempera* consists of pigment particles dispersed in an emulsion of egg yolk (i.e., protein and fatty constituent) and water (i.e., oil-in-water (o/w) emulsion)^6^ (Supporting Information Table S1, Figure S2). The egg yolk contains approximately 50 wt % of dry matter composed of about 31 wt % of lipids, 17 wt % of proteins, and 2 wt % of carbohydrates and ash.^30^ Yolk lipids consist mainly of triacylglycerols (up to 65%), phospholipids (up to 30%), and free cholesterol (4–5%).^31^

Egg yolk has a complex structure consisting of natural nano- (i.e., plasma) and microassemblies (i.e., granulae).^32^ Specifically, (i) granulae, which are circular complexes with diameters ranging from 0.3 to 2 μm, are insoluble protein aggregates primarily composed of high-density lipoproteins (HDLs). HDLs consist of α- and β-lipovitellins, which are glycoconjugates with mannose, galactose, glucosamine, and sialic acid (much higher in α-lipovitellin). (ii) Plasma (i.e., yellow fluid) contains mainly low-density lipoproteins (LDL), which are micelles with diameters ranging from 17 to 60 nm and account for up to 65% of the total yolk proteins as well as soluble proteins.^31,32^

Proteins present in egg yolk exhibit absorption bands due to the characteristic secondary amide group R–CO–NH–R′.^33^ In proteins, the band observed at approximately 1660 cm^–1^ corresponds primarily to the carbonyl (C=O) stretching vibration, characteristic of the amide I mode. In contrast, the band around 1550 cm^–1^ represents the amide II mode, which is a mixed vibration predominantly involving N–H bending (49%) and C–N stretching (29%) within the CO–NH group.

**Figure 3:**
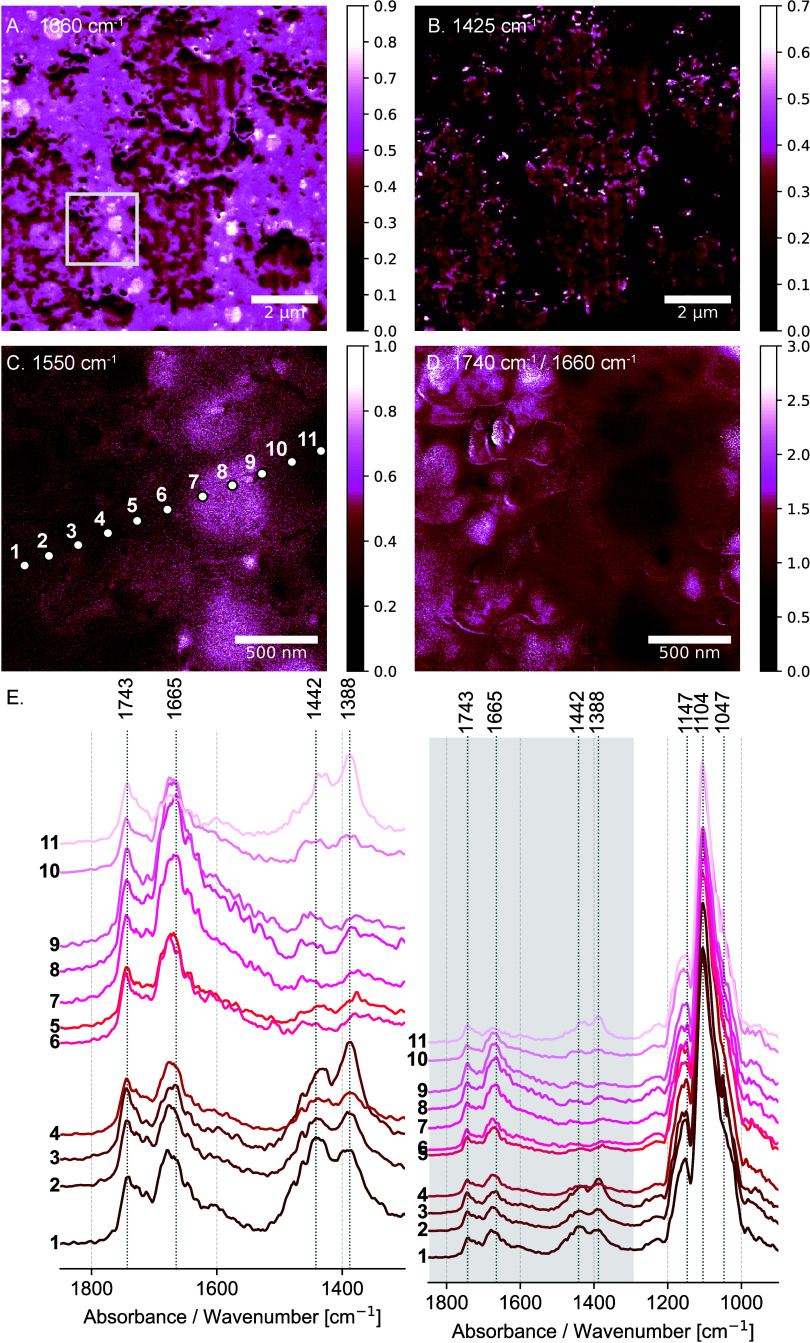
Nanoresolved structure of egg yolk binder in the lead white *tempera* paint layer. (A) The AFM-IR absorption image at 1660 cm^–1^ illustrates the spatial arrangement and shape of HDL-rich granulae within the lead white *tempera* paint layer. (B) The AFM-IR absorption image at 1425 cm^–1^ shows the distribution of lead white pigment particles within the paint layer. (C) Higher-magnification AFM-IR absorption image at 1550 cm^–1^ showing in detail the size and distribution of HDL-rich granulae. (D) The intensity ratio image of the 1740 cm^–1^ to the 1660 cm^–1^ band shows a lower ratio for HDL-rich granulae (indicated in black). (E) The AFM-IR absorption spectra collected at spots 1–11, as shown in (C), show an increased 1665 cm^–1^ signal for HDL-rich granulae, along with higher 1743 cm^–1^ signals for LDL-rich plasma. The spectra are partially obscured by interference from the epoxy embedding medium, particularly in the 1200–1000 cm^–1^ region (Supporting Information Figure S5).

The lipids present in the egg yolk exhibit a carbonyl stretching vibration of the triglyceride ester linkage at 1743 cm^–1^ (Figure 3 E). According to the literature, the intensity ratio of the lipid ester band (1743 cm^–1^) to the amide I band (1660 cm^–1^) varies in HDL and LDL.^34^ This is reflected in the intensity ratio image 1740 cm^–1^/1660 cm^–1^ (Figure 3D). As shown in Figure 3D, the distribution pattern of the lower 1740 cm^–1^ to the 1660 cm^–1^ intensity ratio resembles that of HDL-rich granulae, suggesting the preservation of HDL suspended in LDL-rich plasma within the lead white *tempera* paint layer.

Also, the nanoscale AFM-IR absorption images at 1550 cm^–1^ (Figure 3C) and 1660 cm^–1^ (Figure 3A, Supporting Information Figure S9) suggest the preservation of circular shaped HDL-rich granulae with diameters of ≈300 nm (Figure 3A, B, C) within the lead white *tempera* paint layer. Based on egg yolk reference spectra,^35^ the presence of amide I and amide II bands was anticipated. However, the absence of the amide II band in the AFM-IR spectra (Figure 3E) is unexpected and warrants further investigation to better understand this observation.

The AFM-IR spectra collected at spots 1–4 and 10–11, as indicated in Figure 3C, show notable changes in the spectral region from 1500 cm^–1^ to 1300 cm^–1^ (Figure 3E). Specifically, there is an increase in methylene scissor vibrations and/or methyl asymmetric bending, in addition to the C=O stretching of inorganic carbonate in lead white at 1442 cm^–1^. Furthermore, an increase in the symmetric CH bending vibration of the methyl groups at 1388 cm^–1^ is observed in the areas surrounding the HDL-rich granulae.

The AFM-IR absorption image at 1425 cm^–1^, attributed to the C=O stretch of the inorganic carbonate in lead white, illustrates the arrangement of the pigment particles (Figure 3B). A comparison of the distribution of pigment particles in the oil (Figure 1 D) and *tempera* paint layer (Figure 3B) highlights the disparity in the dispersion of pigment particles related to the paint grinding process: pigment particles of smaller size tend to cluster in *tempera* paint, whereas they are more evenly dispersed in oil paint.

### Tempera Grassa

**Figure 4:**
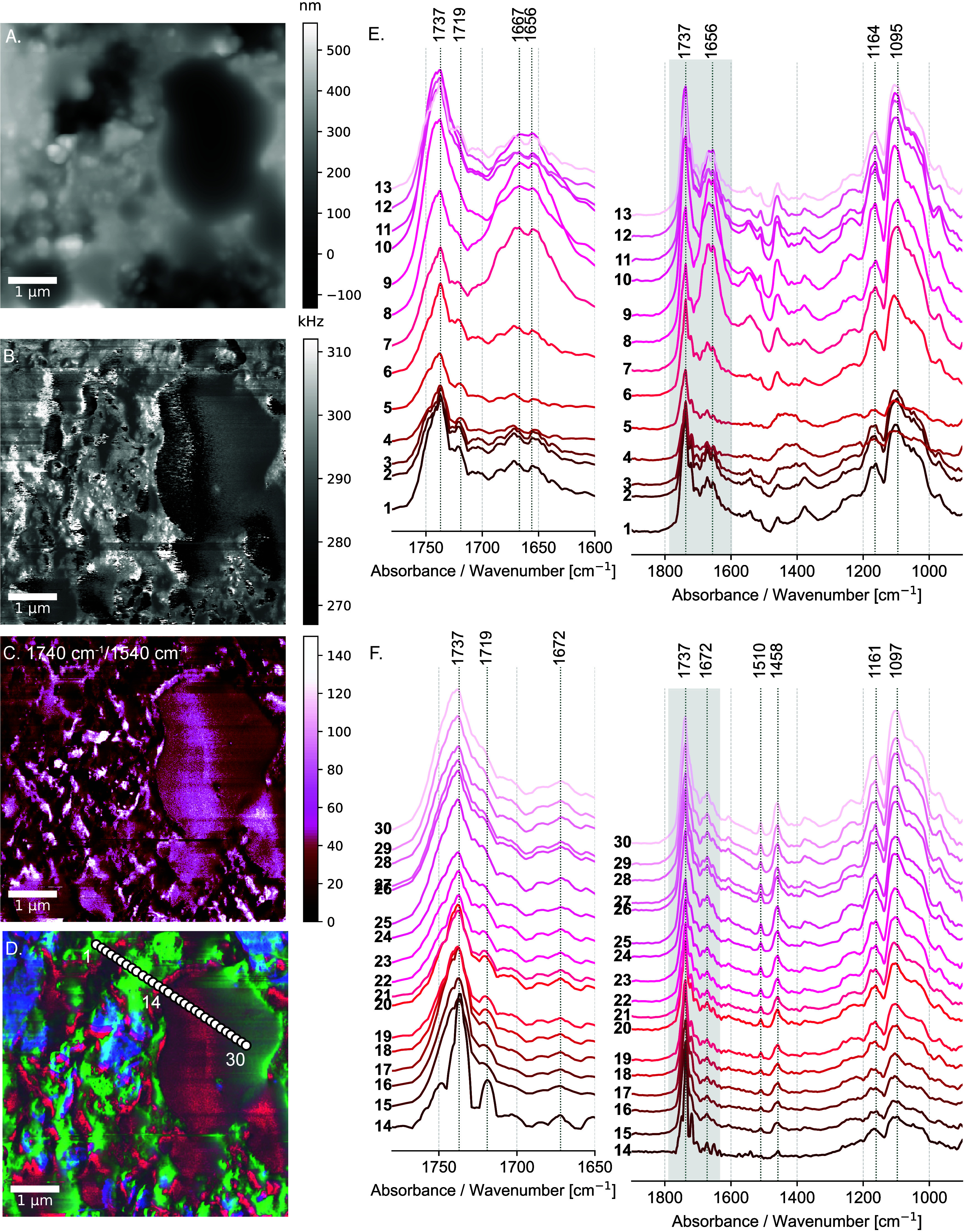
Nanoresolved structure of the lead white *tempera grassa* paint. (A) AFM topographic image of the *tempera grassa* paint layer, showing the distribution of micrometric-size droplets of drying oil. (B) Corresponding phase signal image showing diverse mechanical properties of the paint layer. (C) 1740 cm^–1^/1540 cm^–1^ ratio image highlighting the distribution of the drying oil. (D) False color overlay of the 1740 cm^–1^/1540 cm^–1^ ratio image (in red), 1540 cm^–1^ (in green), and 1420 cm^–1^ (in blue), illustrating the distribution of the drying oil, proteins, and lead white pigment particles, respectively. (E) AFM-IR spectra collected in spots 1–14 (egg yolk region), as indicated in (D); (F) AFM-IR spectra collected in spots 15–30 (drying oil region), as indicated in (D).

In wet paint, *tempera grassa* consists of pigment particles and oil droplets dispersed in a fresh egg yolk and water (o/w) emulsion^6^ (Supporting Information Table S1, Figure S3). The AFM topographic image of *tempera grassa*, shown in Figure 4A, indicates that this structure remains partially intact within the dry paint layer. Oil droplets, varying in size from a few hundred nanometers to 4 μm in diameter, together with pigment particles, form the dispersed phase. This finding is consistent with that of Ranquet et al., whose fluorescence microscopy images of freshly prepared water-based *tempera grassa* paints show the presence of oil droplets of various sizes within the aqueous phase of egg yolk.^6^

The phase signal image (Figure 4B) indicates a contrast between all oil droplets, pointing toward a noncorrected mechanical effect. During scanning in tapping mode, the probe interacts with regions of different mechanical properties, which induce changes in the phase signal. Notably, there is a contrast of the phase signal in all of the oil droplets, indicating changes in the mechanical properties of the drying oil, potentially suggesting an uneven polymerization process within the oil droplets. The analyzed paint layer was prepared 1395 days before this study and underwent artificial aging for 7 days at 40 °C to speed up the curing process. Previous research has shown that *tempera grassa* paints with high oil content can take more than 100 days for the oil curing to commence.^6^ Consequently, extensive oil polymerization is expected to take several years.^6^

The intense band centered at 1737 cm^–1^ is attributed to the carbonyl stretching vibration of the ester groups (COOR) (Figure 4E, F) in triacylglycerols. Triacylglycerols, composed of three fatty acid chains bound to a glycerol molecule, are present both in the egg yolk and in the drying oil; therefore, ester carbonyl stretching cannot be considered diagnostic for the distribution of the drying oil. We use the 1740 cm^–1^/1540 cm^–1^ ratio image, which is expected to be more pronounced in the drying oil, to map its distribution (Figure 4C). The AFM-IR absorption image at 1540 cm^–1^ depicted in green (Supporting Information Figure S10 C) corresponds mainly to the distribution of the in-plane N–H bending vibration and the C–N stretching vibrations of secondary amides in proteins (amide II band). The false color overlay of the 1740 cm^–1^/1540 cm^–1^ ratio image (red), 1540 cm^–1^ (green) and 1420 cm^–1^ (blue) qualitatively shows the distribution of drying oil, proteins, and lead white pigment particles, respectively (Figure 4D, Supporting Information Figure S10). The spatial distribution of oil within the dried paint layer (Figure 4C, D) shows that the oil not only remains intact as micrometer-sized droplets but is also widely distributed within the continuous egg yolk phase. This distribution is linked to the paint drying process: as water evaporates, voids are formed, facilitating the spreading of oil within the continuous egg yolk phase, after paint application and drying.

The band at 1660 cm^–1^ is attributed to the carbonyl stretch C=O of secondary amides (Figure 4E). The combination of carbonyl C=O stretching (1660 cm^–1^) and in-plane N–H bending vibration and/or C–N stretching (1540 cm^–1^) indicates the presence of proteins (see spectra 1–14 (Figure 4E)). This is especially evident at points 7–8 (Figure 4E). The band at 1458 cm^–1^, associated with the methyl and methylene groups, is assigned to the methylene (CH_2_) scissor vibration and the asymmetric bending of the methyl (CH_3_) group. The methyl symmetric bending vibration (umbrella mode) appears at 1376 cm^–1^. The two bands, characteristic of hydrocarbons, are present both in the oil and in egg yolk because of the long hydrocarbon fatty acid chains.

Nanoscale chemical heterogeneity is observed in the egg yolk region (spectra 1–14, Figure 4D, E). The shape and intensity variations of the peaks centered at 1719 cm^–1^ and 1737 cm^–1^ are evident. The chemical processes involved in oil curing remain elusive when occurring in the presence of proteins. The uneven distribution of carbonyl groups may serve as compelling evidence that proteins mediate this distinct chemistry, leading to the formation of diverse species at the interface and at the protein level. It might also be related to the variation of the secondary structures of the protein chains in space because of the proximity of different types of interface.

Closer inspection of the spectra relative to the oil region shown in Figure 4F shows intensity and shape differences of the carbonyl band at 1739 cm^–1^ and 1719 cm^–1^, also emphasizing the nanoscale chemical heterogeneity along the drying oil droplets. The band centered at 1719 cm^–1^ is an overlap of the absorption bands attributed to the C=O stretch in carboxylic acid (COOH), aldehyde (CHO), and ketone (COC) groups.

The out-of-plane =C–H bending vibrations show up at 968 cm^–1^ and indicate the presence of *trans* alkenes, which are formed upon curing. The clear detection of unreacted unsaturations within the oil droplets is an indication that curing is still incomplete. Compared to the oil and *tempera* paint layers discussed above, the contamination of epoxy resin is less prominent in the spectra of the *tempera grassa* paint layer. This is due to the additional curing time of the embedding medium. We used an ultramicrotome to flatten the cross-sectional surface three months after embedding.

### Insights into Chemical Heterogeneity of Paints

A comprehensive understanding of paint composition requires insight into both the chemical heterogeneity and the spatial organization of its constituents. Our findings show that, in traditional painting techniques, such as tempera and oil painting, heterogeneity arises from drying, aging, or the intrinsic submicrometer structures of individual components. In mixed-media paints, the spatial distribution of binder components provides additional valuable insights into their preparation. Although spectroscopic analyses yield an average composition of materials, advanced imaging techniques such as AFM-IR, with nanoscale resolution, can reveal localized heterogeneity and specific organizational patterns.

Accurate identification of paint binders, a critical first step in heritage studies, is particularly challenging in mixed-media paints, where proteins and oils coexist within a single layer. Our findings demonstrate that nanoscale imaging techniques significantly facilitate this process by probing the spatial distribution of proteins and oils at the submicroscopic scale. Mapping these distributions not only identifies the components used but also reveals their microstructural organization, which is critical for understanding the original paint formulation. In addition, this approach ensures that the detected lipids and proteins are intrinsic to the paint binder itself, helping to prevent misinterpretations caused by contaminants from the underlying preparation layers, prior conservation treatments, or separate paint layers.

In *tempera grassa*, the binder exemplifies a highly heterogeneous medium, where pigment particles are simultaneously exposed to proteins and lipids from linseed oil and egg yolk. The intricate chemical interactions within a lipid–protein binder in these paints as well as the role of pigment particles in influencing these processes remain areas that require further study. The addition of a drying oil to a proteinaceous binder can shift oxidative damage from lipids to proteins through a process known as cooxidation.^36^ Lipid oxidation generates four primary classes of reactive species: free radicals, hydroperoxides, epoxides, and secondary carbonyl compounds.^36^ These species interact with nonlipid molecules, propagating oxidative damage from lipids to proteins through co-oxidation, a highly complex process that remains understudied in this context. Understanding these interactions is essential for studying the long-term behavior of mixed-media paints.

Continued research into the submicroscopic behavior of artists’ paint layers is crucial for deepening our knowledge of degradation processes and has the potential to reveal historical artistic practices. It provides unique insights into the techniques of the Old Masters, illuminating the methods they used to create their masterpieces.

## Conclusion

This study aimed to gain a comprehensive understanding of the nanostructure and chemistry of organic binders in paint layers and relate them to the paint composition and formulation. Nanoinfrared imaging provides an advanced method for visualizing the chemical heterogeneity of complex organic systems. The integration of chemical information with nanoresolved spatial data enables visualization and interpretation of not only the original chemistry and structure but also the reaction pathways driving heterogeneity, such as the preferential accumulation of polar groups near lead white pigment particles in the oil paint layer.

The findings demonstrate nanoscale heterogeneity in two historically significant painting techniques: the preservation of submicrometer structures in *tempera* paint and the chemical inhomogeneity within oil paint. Furthermore, the study provides significant information about the structure of the mixed-media paint *tempera grassa*. However, the chemistry of paint film formation and aging of mixed-media paints remain areas that require further investigation. We now have evidence of the effects of different paint formulations and preparation processes on paint microstructures; yet, these findings still need to be correlated with the paintings of Old Masters. The current investigation establishes a framework for exploring Renaissance masterpieces at the nanoscale, unlocking a new level of understanding of their material composition and technological craftsmanship.

## Supporting Information Available

Detailed descriptions of experimental and analytical methods, including a description of materials, optical microscopy images of the samples, instrumentation details, and AFM-IR data processing; AFM-IR spectrum of the embedding epoxy resin, as well as additional AFM-IR absorption, topographic, and phase signal images of the samples (PDF)

## References

[ref1] Cotte M.; Genty-Vincent A.; Janssens K.; Susini J. Applications of synchrotron X-ray nano-probes in the field of cultural heritage. Comptes Rendus. Physique 2018, 19, 575–588. 10.1016/j.crhy.2018.07.002.

[ref2] Dunkerton J. Modifications to traditional egg tempera techniques in fifteenth century Italy. In Early Italian Paintings: Techniques and Analysis; 1996; pp 29–34.

[ref3] DeGhetaldi K. From egg to oil: the early development of oil painting during the Quattrocento. Ph.D. Thesis, University of Delaware, 2016.

[ref4] Dietemann P.; Fischer U.; Karl D. Florentiner Malerei – Alte Pinakothek; Die Gemälde des 14. bis 16. Jahrhunderts; Deutscher Kunstverlag, 2017; pp 92–105.

[ref5] Thillaye du Boullay C.; Jaber M.; Le Denic M.; Gerony F.; Bordes R.; Meriguet G.; Rollet A.-L.; Walter P.; de Viguerie L. On the way to tempera grassa: unraveling the properties of emulsion-based paint binders. Colloids Surf., A 2023, 673, 131816 10.1016/j.colsurfa.2023.131816.

[ref6] Ranquet O.; Duce C.; Caroti G.; Dietemann P.; Bonaduce I.; Willenbacher N. Tempera and Tempera Grassa From Wet Paints to Solid Films. ACS Appl. Polym. Mater. 2023, 5, 4664–4677. 10.1021/acsapm.3c00179.

[ref7] Ranquet O.; Duce C.; Bramanti E.; Dietemann P.; Bonaduce I.; Willenbacher N. A holistic view on the role of egg yolk in Old Masters’ oil paints. Nat. Commun. 2023, 14, 1534 10.1038/s41467-023-36859-5. 36977659 PMC10050151

[ref8] Colombini M. P.; Andreotti A.; Bonaduce I.; Modugno F.; Ribechini E. Analytical strategies for characterizing organic paint media using gas chromatography/mass spectrometry. Accounts of chemical research 2010, 43, 715–727. 10.1021/ar900185f. 20180544

[ref9] Dallongeville S.; Garnier N.; Rolando C.; Tokarski C. Proteins in art, archaeology, and paleontology: from detection to identification. Chem. Rev. 2016, 116, 2–79. 10.1021/acs.chemrev.5b00037. 26709533

[ref10] Geddes da Filicaia E.; Evershed R. P.; Peggie D. A. Review of recent advances on the use of mass spectrometry techniques for the study of organic materials in painted artworks. Anal. Chim. Acta 2023, 1246, 340575 10.1016/j.aca.2022.340575. 36764767

[ref11] Bouvier C.; Van Nuffel S.; Walter P.; Brunelle A. Time-of-flight secondary ion mass spectrometry imaging in cultural heritage: A focus on old paintings. Journal of Mass Spectrometry 2022, 57, e4803 10.1002/jms.4803. 34997666

[ref12] Leona M.; Stenger J.; Ferloni E. Application of surface-enhanced Raman scattering techniques to the ultrasensitive identification of natural dyes in works of art. J. Raman Spectrosc. 2006, 37, 981–992. 10.1002/jrs.1582.

[ref13] Vandenabeele P.; Edwards H. G.; Moens L. A decade of Raman spectroscopy in art and archaeology. Chem. Rev. 2007, 107, 675–686. 10.1021/cr068036i. 17315936

[ref14] Prati S.; Joseph E.; Sciutto G.; Mazzeo R. New advances in the application of FTIR microscopy and spectroscopy for the characterization of artistic materials. Accounts of chemical research 2010, 43, 792–801. 10.1021/ar900274f. 20476733

[ref15] Capitani D.; Di Tullio V.; Proietti N. Nuclear magnetic resonance to characterize and monitor cultural heritage. Prog. Nucl. Magn. Reson. Spectrosc. 2012, 64, 29–69. 10.1016/j.pnmrs.2011.11.001. 22578316

[ref16] Keune K.; Boon J. J. Analytical imaging studies of cross-sections of paintings affected by lead soap aggregate formation. Studies in conservation 2007, 52, 161–176. 10.1179/sic.2007.52.3.161.

[ref17] Mazzeo R.; Prati S.; Quaranta M.; Joseph E.; Kendix E.; Galeotti M. Attenuated total reflection micro FTIR characterisation of pigment–binder interaction in reconstructed paint films. Anal. Bioanal. Chem. 2008, 392, 65–76. 10.1007/s00216-008-2126-5. 18454281

[ref18] Boon J. J.; Keune K.; Van der Weerd J.; Geldof M.; Van Asperen de Boer J.R.J. Imaging microspectroscopic, secondary ion mass spectrometric and electron microscopic studies on discoloured and partially discoloured smalt in cross-sections of 16th century paintings. Chimia 2001, 55, 952–952. 10.2533/chimia.2001.952.

[ref19] Dazzi A.; Prater C. B. AFM-IR: Technology and applications in nanoscale infrared spectroscopy and chemical imaging. Chem. Rev. 2017, 117, 5146–5173. 10.1021/acs.chemrev.6b00448. 27958707

[ref20] Morsch S.; Van Driel B. A.; van den Berg K. J.; Dik J. Investigating the photocatalytic degradation of oil paint using ATR-IR and AFM-IR. ACS Appl. Mater. Interfaces 2017, 9, 10169–10179. 10.1021/acsami.7b00638. 28256818

[ref21] Ma X.; Beltran V.; Ramer G.; Pavlidis G.; Parkinson D. Y.; Thoury M.; Meldrum T.; Centrone A.; Berrie B. H. Revealing the Distribution of Metal Carboxylates in Oil Paint from the Micro-to Nanoscale. Angew. Chem. 2019, 131, 11778–11782. 10.1002/ange.201903553. PMC9798385 31226237

[ref22] Ma X.; Pavlidis G.; Dillon E.; Beltran V.; Schwartz J. J.; Thoury M.; Borondics F.; Sandt C.; Kjoller K.; Berrie B. H. Micro to Nano: multiscale IR analyses reveal zinc soap heterogeneity in a 19th-century painting by Corot. Anal. Chem. 2022, 94, 3103–3110. 10.1021/acs.analchem.1c04182. 35138807

[ref23] Dazzi A.; Mathurin J.; Leclere P.; Nickmilder P.; De Wolf P.; Wagner M.; Hu Q.; Deniset-Besseau A. Photothermal AFM-IR Depth Sensitivity: An Original Pathway to Tomographic Reconstruction. Anal. Chem. 2024, 96, 17931 10.1021/acs.analchem.4c01969. 39483054

[ref24] Kebukawa Y.; Mathurin J.; Dartois E.; Dazzi A.; Deniset-Besseau A.; Duprat J.; Remusat L.; Noguchi T.; Miyake A.; Igami Y.; Paoletti M. V.; Zolensky M. E.; Engrand C.; Sandt C.; Borondics F.; Yamashita S.; Wakabayashi D.; Takeichi Y.; Takahashi Y.; et al. Complex mixture of organic matter in a xenolithic clast from the Zag meteorite revealed by coordinated analyses using AFM-IR, NanoSIMS and STXM/XANES. Icarus 2023, 400, 115582 10.1016/j.icarus.2023.115582.

[ref25] Pizzimenti S.; Bernazzani L.; Tine M. R.; Treil V.; Duce C.; Bonaduce I. Oxidation and cross-linking in the curing of air-drying artists’ oil paints. ACS Appl. Polym. Mater. 2021, 3, 1912–1922. 10.1021/acsapm.0c01441.

[ref26] Tumosa C. S.; Mecklenburg M. F. The influence of lead ions on the drying of oils. Studies in Conservation 2005, 50, 39–47. 10.1179/sic.2005.50.Supplement-1.39.

[ref27] de Viguerie L.; Payard P.; Portero E.; Walter P.; Cotte M. The drying of linseed oil investigated by Fourier transform infrared spectroscopy: Historical recipes and influence of lead compounds. Prog. Org. Coat. 2016, 93, 46–60. 10.1016/j.porgcoat.2015.12.010.

[ref28] Meilunas R. J.; Bentsen J. G.; Steinberg A. Analysis of aged paint binders by FTIR spectroscopy. Studies in conservation 1990, 35, 33–51. 10.1179/sic.1990.35.1.33.

[ref29] Hermans J.; Zuidgeest L.; Iedema P.; Woutersen S.; Keune K. The kinetics of metal soap crystallization in oil polymers. Phys. Chem. Chem. Phys. 2021, 23, 22589–22600. 10.1039/D1CP03479K. 34591054 PMC8514046

[ref30] Stadelman W.; Cotterill O. Egg Science and Technology; Professional Bks; Avi Publishing Company, 1986.

[ref31] Sunwoo H. H.; Gujral N. Handbook of food chemistry; Springer: Berlin, Heidelberg, 2015; pp 331–363.

[ref32] Anton M. Egg yolk: structures, functionalities and processes. Journal of the Science of Food and Agriculture 2013, 93, 2871–2880. 10.1002/jsfa.6247. 23716191

[ref33] Socrates G. Infrared and Raman characteristic group frequencies: tables and charts; John Wiley & Sons, 2004.

[ref34] Krilov D.; Balarin M.; Kosović M.; Gamulin O.; Brnjas-Kraljević J. FT-IR spectroscopy of lipoproteins—A comparative study. Spectrochimica Acta Part A: Molecular and Biomolecular Spectroscopy 2009, 73, 701–706. 10.1016/j.saa.2009.03.015. 19414281

[ref35] Gerony F.; de Viguerie L.; Boulard Y.; Thillaye du Boullay C.; Michot L.; Rollet A.-L.; Meriguet G.; Jaber M. Monitoring the aging process in egg-tempera paint films. Spectrochimica Acta Part A: Molecular and Biomolecular Spectroscopy 2025, 328, 125371 10.1016/j.saa.2024.125371. 39579723

[ref36] Schaich K. M. Lipid oxidation: theoretical aspects. Bailey’s industrial oil and fat products 2005, 1, 273–303. 10.1002/047167849X.bio067.

