# Supplementary material for: Nanoscale Probing of the Organic Binder in Artists’ Paint Layers: Organic Phases and Chemical Heterogeneity
Source: ACS Appl Mater Interfaces. 2025 Jan 24;17(5):8464–73. doi: 10.1021/acsami.4c16430 (PMC11803564; doi:10.1021/acsami.4c16430)
Supplement: Supplementary file 1 — am4c16430_si_001.pdf [file am4c16430_si_001.pdf]

Supporting Information

Nanoscale Probing of the Organic Binder in Artists' Paint Layers: Organic Phases and Chemical Heterogeneity

Rafaella Georgiou,[1,2,*] Alexandre Dazzi,[3] Jeremie Mathurin,[3] Celia Duce,[1] Patrick Dietemann,[4] Mathieu Thoury,[5] Ilaria Bonaduce[1]

[1] Department of Chemistry and Industrial Chemistry, Università di Pisa, via Moruzzi 13, 56124 Pisa, Italy

[2] Department of Physics, University of Wisconsin-Madison, 1150 University Avenue, 53706 Madison, Wisconsin, United States

[3] Institut de Chimie Physique, UMR8000, Université Paris-Saclay, CNRS, 91405 Orsay, France

[4] Bayerische Staatsgemäldesammlungen, Doerner Institut, Richard-Wagner-Str. 1, 80333 Munich, Germany

[5] CNRS, ministère de la Culture, UVSQ, MNHN, UAR3461, Université Paris-Saclay, Institut photonique d'analyse non-destructive européen des matériaux anciens, 91192 Saint-Aubin, France

*Email: rafaella.georgiou@dcci.unipi.it

Abstract: Understanding paint structures at the nanoscopic level can address key questions related to artistic techniques, paint formulation, and long-term preservation of artworks. This involves examining the spatial chemical complexity, the formation of molecular networks, and interactions between organic and inorganic constituents. Depending on the paint preparation methods, proteins and drying oils - the most common binders in traditional artistic practices - can be integrated to produce paints with diverse structures and nanoscale chemical intricacies. In this study, we utilize atomic force microscopy-based infrared spectroscopy (AFM-IR) to investigate the spatial chemical complexity and reaction pathways of organic species in artists' paints, including oil, *tempera* and mixed-media *tempera grassa*. By analyzing these paints at the nanoscale, we established connections between their structural organization, chemistry and formulation.

Table of Contents

1. Experimental Procedures *3*

1.1. Materials and Paint Preparation **3**

1.1.1. Aging of Paint Layers **3**

1.1.2. Stratigraphy Exposure and Surface Preparation **3**

1.2. Atomic Force Microscopy-Based Infrared Spectroscopy (AFM-IR) **5**

2. Embedding Resin *7*

3. Topographic, Phase-Signal, and AFM-IR Absorption Maps *8*

4. References *11*

1. Experimental Procedures

1.1. Materials and Paint Preparation

The model paints were thoroughly prepared by Ophélie Ranquet using lead white sourced from Kremer Pigmente (Germany) and synthetic ultramarine blue (Blu Oltramare Puro M (6018)) from Abralux Colori Beghè (Italy). Fresh hen egg yolk and cold pressed linseed oil (Maimeri, Italy) were used as paint binders (Ranquet, 2023).

- **Oil Paint.** To prepare the lead white and ultramarine blue oil paints, we mixed the selected pigment with cold pressed linseed oil. The process involved grinding the pigment with a flat muller on a glass plate, gradually incorporating the oil until a smooth consistency was achieved. Initially, a stiff dry paste formed which progressively softened with continued grinding, eventually achieving the desired texture of the paint.
- ***Tempera* Paint.** For *tempera* paint, lead white was combined with fresh egg yolk and distilled water. The mixture was ground on a glass plate using a flat muller to achieve a well-dispersed paint. As *tempera* dries quickly, distilled water was intermittently added during the grinding process to ensure a sufficient grinding time.
- ***Tempera Grassa.*** *Tempera grassa* was prepared by gradually adding drops of linseed oil to a freshly prepared tempera paint (egg yolk, pigment, and distilled water), while continuing to grind the mixture with a muller.

The freshly prepared paints were carefully applied to the glass slides as thin, even layers using a palette knife and then allowed to dry under ambient conditions. The volume compositions of the dried paint are summarized in Table S1.

Table S1. Composition of oil, tempera, and *tempera grassa* paints (in vol %).

Sample		Pigment	Egg yolk dried matter (EY)	Linseed oil (LO)	Distilled water
LWLO[1]		32	-	68	-
LWEY[2]	wet	16	29	-	55
	dry	36	64	-	-
LWTG[3]	wet	13	24	17	46
	dry	24	45	31	-
UBLO[4]		32	-	68	-

1.1.1. Aging of Paint Layers

- The layers of lead white oil and *tempera* paint naturally aged for approximately 45 months prior to analysis.
- The lead white *tempera grassa* paint layer was aged naturally for approximately 46 months. The embedded cross section was subjected to additional artificial aging in an oven set at 40 °C for 7 days.
- The ultramarine blue oil paint layer was aged for approximately 14 months prior to analysis.

1.1.2. Stratigraphy Exposure and Surface Preparation

Microsamples were collected from each of the aged paint layers and embedded in EpoFix epoxy resin supplied by Agar Scientific (U.K.). The embedded samples were subjected to microtoming, which exposed the stratigraphy of the samples with minimal surface roughness. This process produced smooth cross-sectional surfaces, depicted in Figure S1, Figure S2, Figure S3, Figure S4.

[1] lead white (LW) linseed oil (LO)
[2] lead white (LW) egg yolk (EY)
[3] lead white (LW) *tempera grassa* (TG)
[4] ultramarine blue (UB) linseed oil (LO)

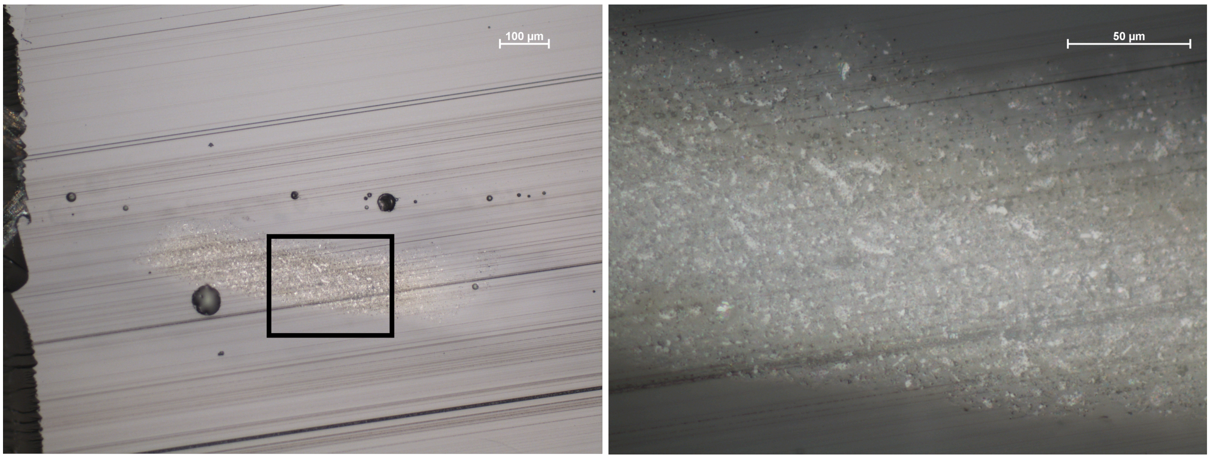

Figure S1. Optical microscopy image of the examined cross section collected from the lead white oil paint layer (LWLO, Table S1).

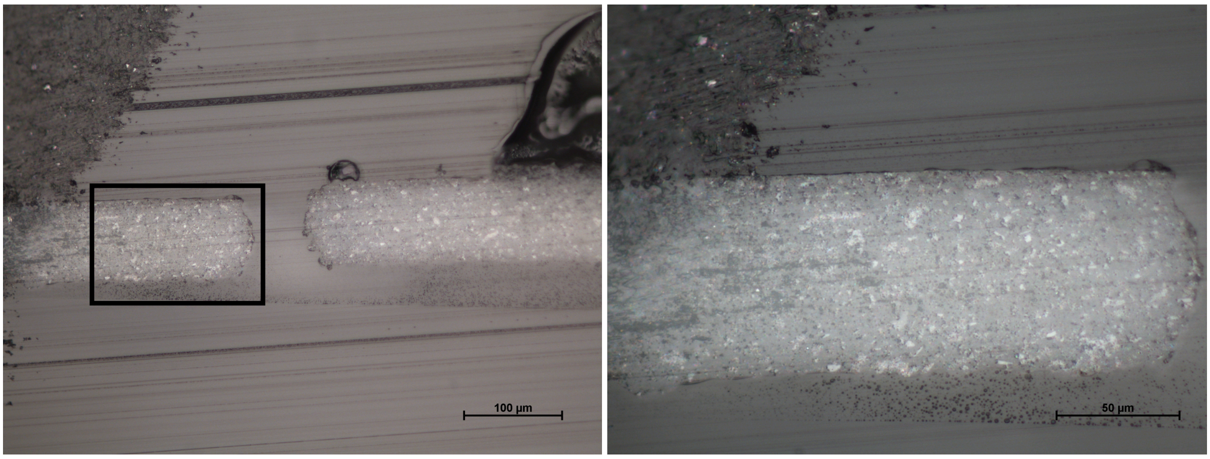

Figure S2. Optical microscopy image of the examined cross section collected from the lead white *tempera* paint layer (LWEY, Table S1).

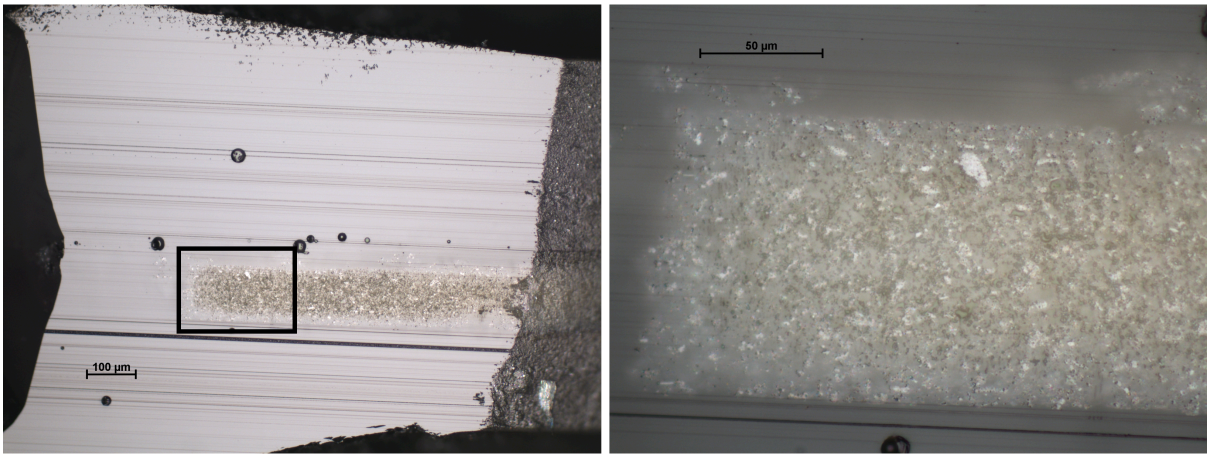

Figure S3. Optical microscopy image of the examined cross section collected from the lead white *tempera grassa* paint layer (LWTG, Table S1).

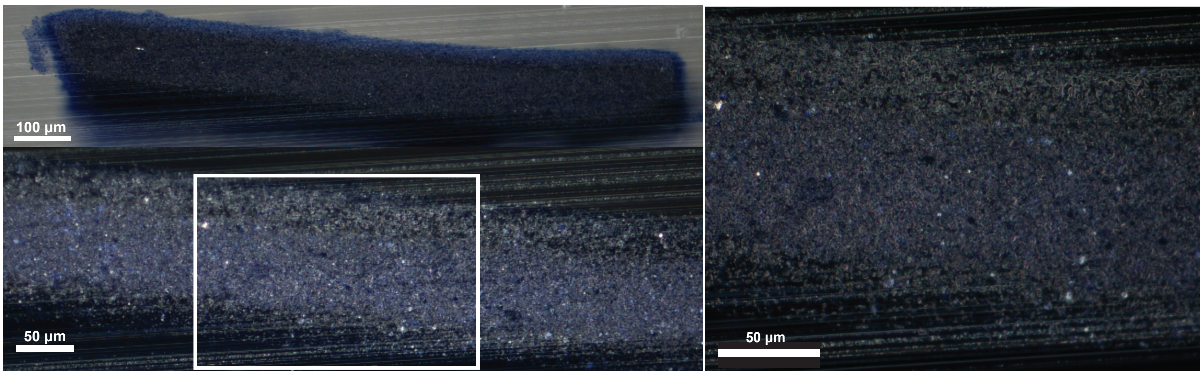

Figure S4. Optical microscopy image of the examined cross section collected from the synthetic ultramarine blue oil paint (UBLO, Table S1).

1.2. Atomic Force Microscopy-Based Infrared Spectroscopy (AFM-IR)

AFM-IR measurements were conducted at the Institut de Chimie Physique (ICP) at Université Paris-Saclay using a Bruker IconIR. This system integrates a tunable infrared (IR) laser, covering the mid-IR range from 900 cm^{-1} to 1900 cm^{-1}, with an atomic force microscope (AFM). The IR laser pulse reaches the sample surface, and it is absorbed by the sample, causing a localized heating expansion detected with the AFM probe. The magnitude of the mechanical force is proportional to the absorption coefficient, which is used to generate an IR spectrum.

Tapping Mode Scanning. The samples were scanned in tapping mode to simultaneously collect topographic images and IR maps at a fixed laser wavenumber. For these experiments, we used a Nanosensors cantilever model PPP-NCHAu-MB, with a stiffness of 50 N/m and a resonance frequency of 270 kHz. The difference-frequency detection scheme was used, with the IR signal captured using a secondary tapping mode at 270 kHz while the drive frequency was maintained at 1.7 MHz. This resulted in a laser repetition rate of 1.43 MHz. A Phase Locked Loop (PLL) scheme was used to compensate for mechanical effects on the resonance, using the following parameters: P = 0.1, I = 0.1, frequency minimum = -20 kHz and frequency maximum = +20 kHz. AFM-IR maps and spectra were collected using the same cantilever with identical characteristics.

Phase Locked Loop (PLL) Measurements. AFM-IR measurements are performed using tapping mode, where the cantilever is driven to maintain a constant oscillation. For each oscillation mode, two channels are recorded: one corresponding to the amplitude of the oscillation and the other to its phase. Since the tapping mode is highly sensitive to interactions between the tip and the sample surface, the frequency of oscillation modes evolves as the tip scans the surface, responding to mechanical changes in the sample.

Working with a fixed laser repetition rate under these conditions would result in erroneous contrast in the IR mapping. To address this, a phase-locked loop (PLL) is integrated into the system to continuously adjust and match these frequency changes. At each point, the PLL applies a correction to maintain the maximum amplitude of oscillation, which corresponds to an inflection point in the phase signal. This ensures accurate tracking of frequency shifts and precise IR mapping.

Simultaneously, the phase changes applied by the PLL are recorded. Since these corrections are directly related to the mechanical interactions between the tip and the sample, the resulting phase image provides valuable information on local variations in the mechanical properties of the sample.

AFM-IR Spectra Collection. For the collection of AFM-IR spectra, the tip was held at a specific position, while the tunable IR laser was adjusted to different wavenumbers to capture spectral data with a spectral resolution of 1 cm^{-1}. The AFM-IR spectra are interpreted similarly to Fourier-transform infrared spectroscopy (FTIR) spectra.

Data Processing of AFM-IR Absorption Maps. Data processing for the AFM-IR absorption maps was carried out using Python. We normalized each absorption map at a specific wavenumber by subtracting the minimum pixel intensity (x_{min}) from each pixel (x) and dividing by the range of pixel intensities ($x_{max} - x_{min}$) :

$$(x - x_{min})/(x_{max} - x_{min})$$

As a result, all the pixel intensity values in the normalized images are scaled to a range between 0 and 1. This ensures that the images are uniformly scaled and suitable for comparative analysis across different wavenumbers.

Generation of RGB Images. The generation of RGB images was carried out using Python. To create RGB images (Fig.1D, Fig.4C), each individual channel was independently standardized by subtracting the mean value of the dataset (μ) from the original value (x) and dividing by the standard deviation of the dataset (σ):

$$Z = \frac{x - \mu}{\sigma}$$

$$\mu = \frac{1}{N}\sum_{i=1}^{N} x_i$$

$$\sigma = \sqrt{\frac{1}{N}\sum_{i=1}^{N} (x_i - \mu)^2}$$

The normalized channels are then merged to create an RGB image.

2. Embedding Resin

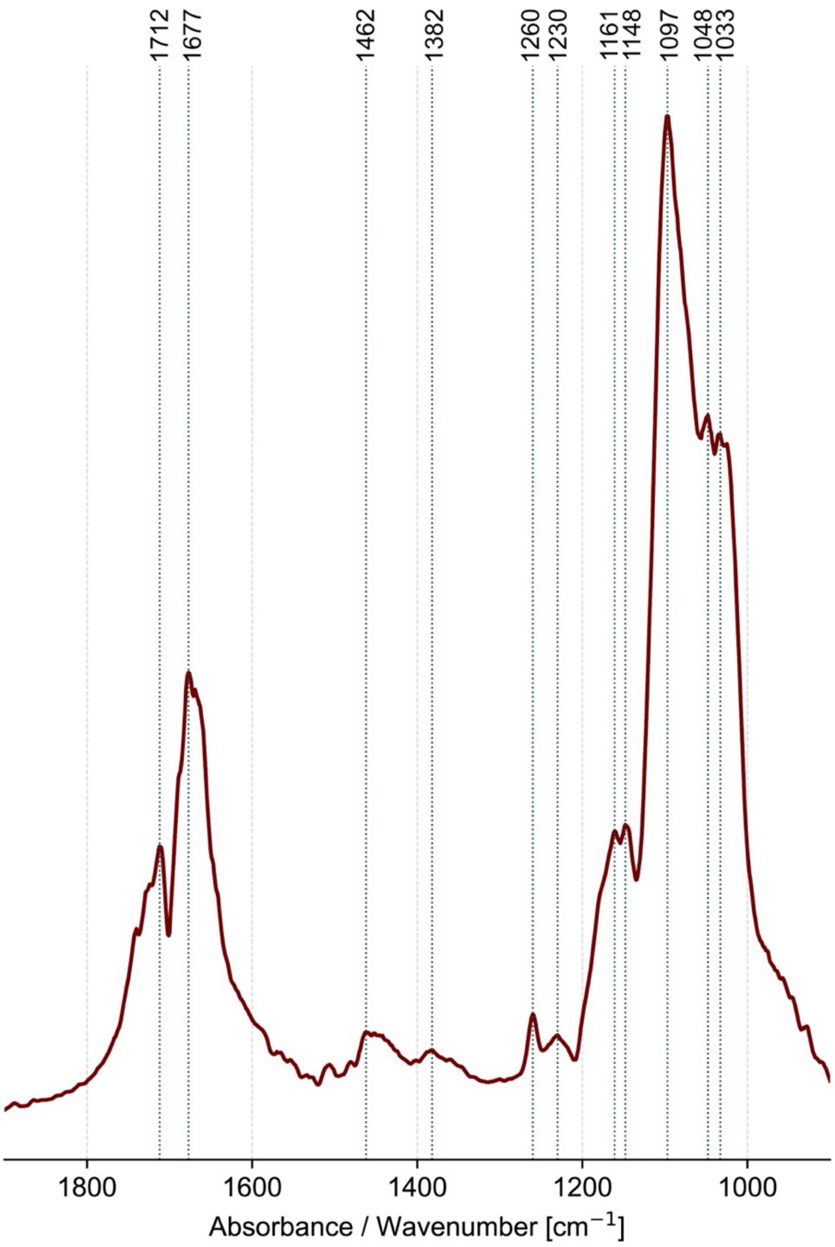

Figure S5. AFM-IR spectrum of embedding epoxy resin (EpoFix supplied by Agar Scientific (United Kingdom)).

3. Topographic, Phase-Signal, and AFM-IR Absorption Maps

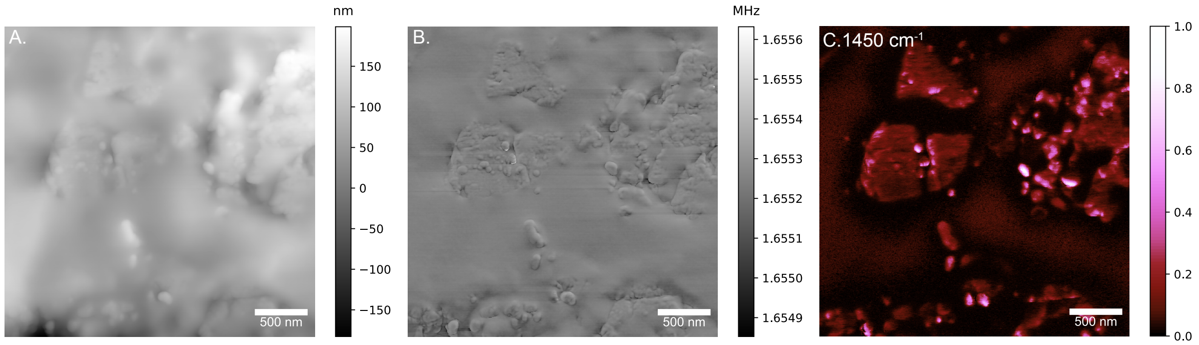

Figure S6. (A) AFM topographic image of the lead white oil paint layer; (B) Phase signal image of the lead white oil paint layer, highlighting regions with different mechanical properties indicating the distribution of lead white particles and linseed oil; (C) AFM-IR absorption image at 1450 cm^{-1} of the lead white oil paint layer (LWLO, Table S1, Figure S1).

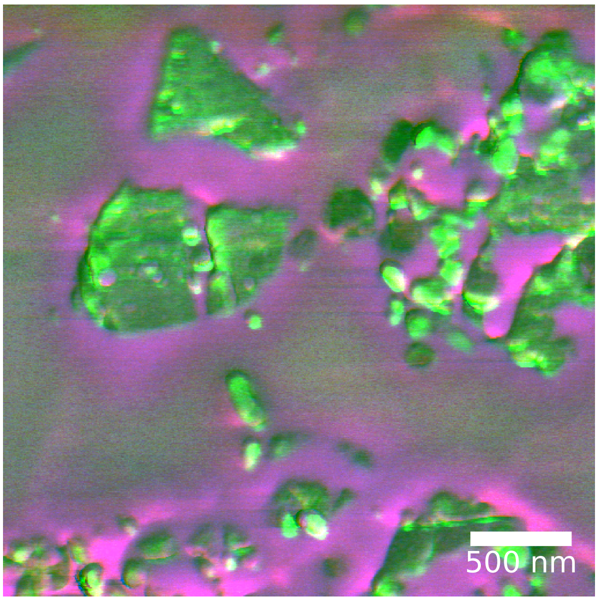

Figure S7. A qualitative false color overlay of 1740 cm^{-1} (red), 1450 cm^{-1} (green) and 1710 cm^{-1} (blue) AFM-IR absorption images highlights the strong absorption of polar groups (e.g., esters and other carbonyl groups) around the lead white pigment particles.

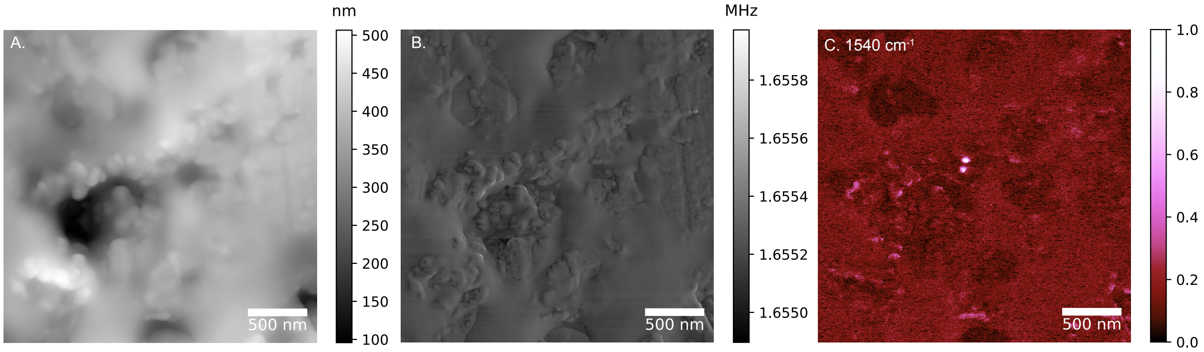

Figure S8. (A) AFM topographic image of lead white oil paint layer; (B) Phase signal image of lead white oil paint layer highlighting regions with different mechanical properties indicating the distribution of lead white particles and linseed oil; (C) AFM-IR absorption image at 1540 cm^{-1} showing the formation of lead soaps of nanometric size in the lead white oil paint layer (LWLO, Table S1, Figure S1).

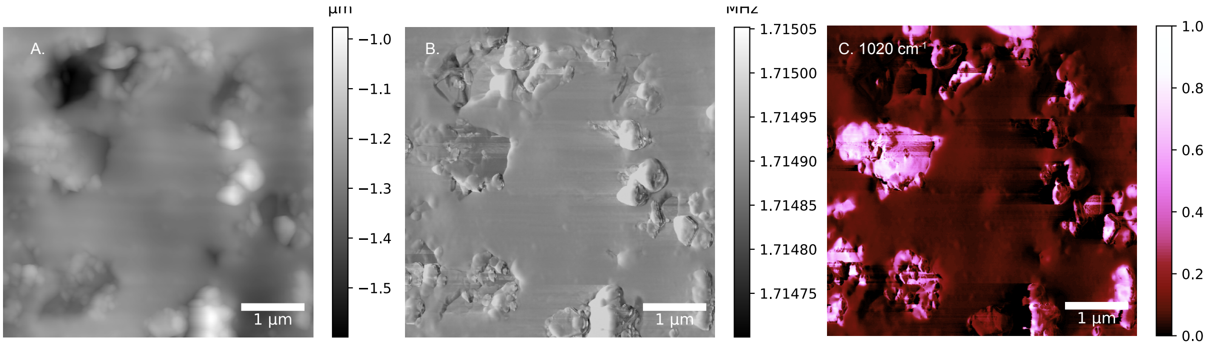

Figure S9. *(*A) AFM topographic image of the synthetic ultramarine blue oil paint layer; (B) Phase signal image of the synthetic ultramarine blue oil paint layer highlighting regions with different mechanical properties indicating the distribution of ultramarine blue particles and linseed oil; *(*C) AFM-IR absorption image at 1020 cm^{-1} of the synthetic ultramarine blue oil paint layer showing the distribution of the pigment particles (UBLO, Figure S4, Table S1).

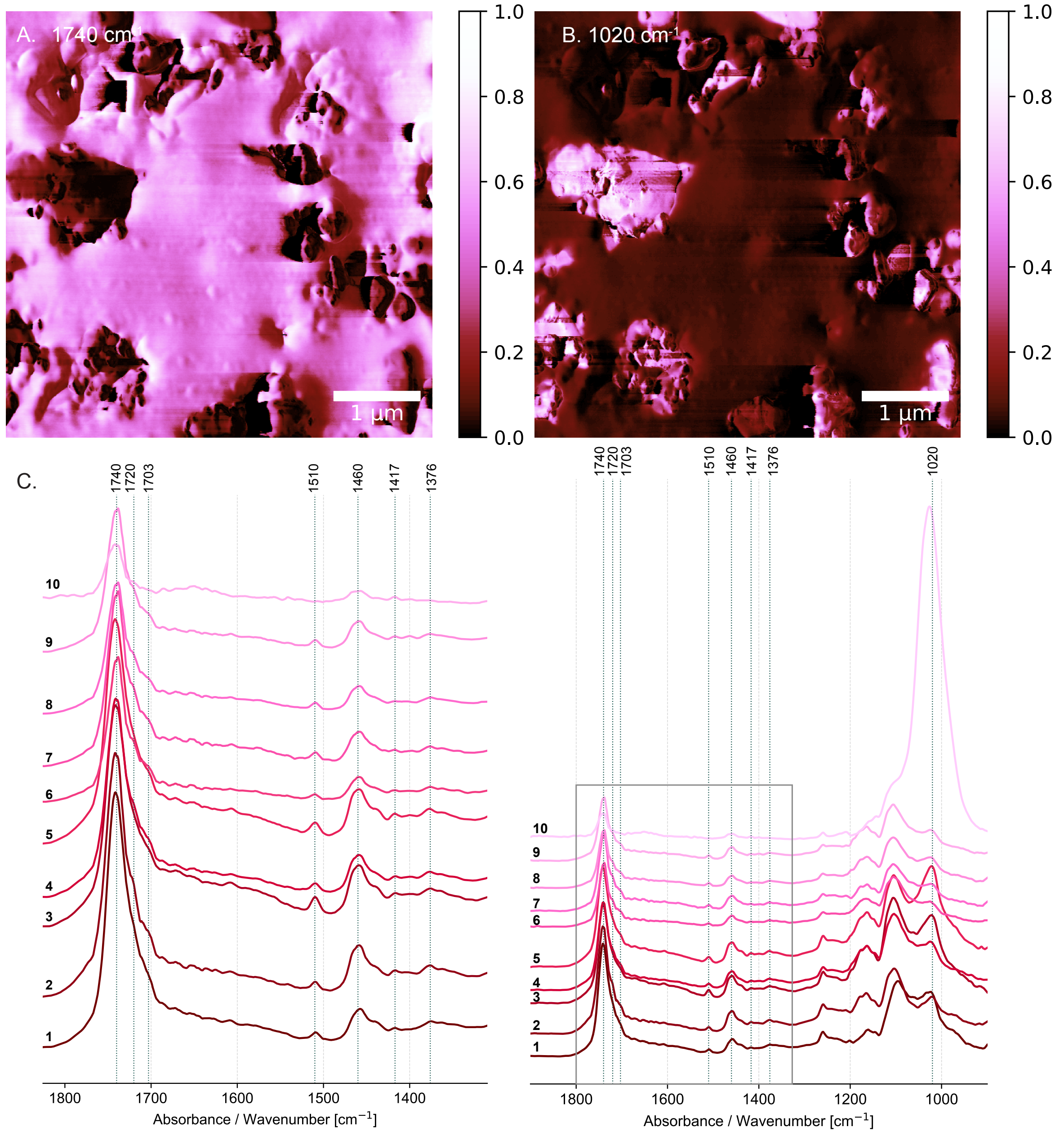

Figure S10. Nanoresolved structure of the synthetic ultramarine blue oil paint layer. (A) AFM-IR absorption image at 1740 cm^{-1} showing the distribution of ester groups in oil binder; (B) AFM-IR absorption image at 1020 cm^{-1} showing the distribution of the synthetic ultramarine blue particles; (C) AFM-IR spectra collected at spots 1-10 indicated in Figure *S12 A*.

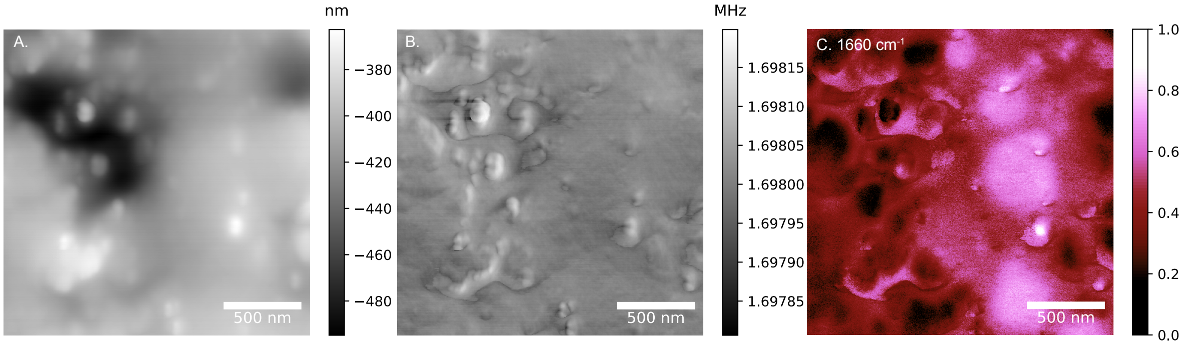

Figure S11. (A) AFM topographic image of the lead white egg yolk *tempera* paint layer; (B) Phase signal image of the lead white egg yolk *tempera* paint layer highlighting regions of different mechanical properties; (C) AFM-IR absorption image at 1660 cm^{-1} of the lead white egg yolk *tempera* paint layer (LWEY, Table S1, Figure S2).

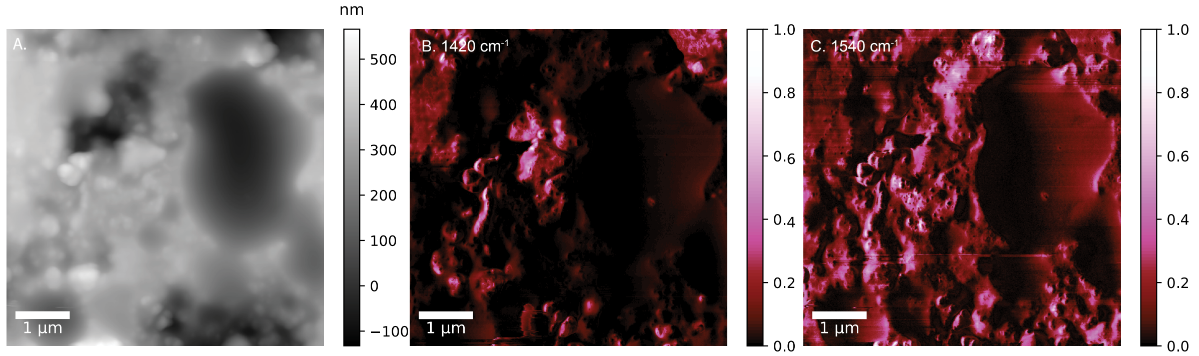

Figure S12. (A) AFM topographic image of the lead white *tempera grassa* paint layer; (B) AFM-IR absorption image at 1420 cm^{-1} of the lead white *tempera grassa* paint layer; (C) AFM-IR absorption image at 1540 cm^{-1} of the lead white *tempera grassa* paint layer (LWEY, Table S1, Figure S3).

4. References

[1] Ranquet Ophélie, D. C. (2023). A holistic view on the role of egg yolk in Old Masters' oil paints. *Nature Communications*, 1534.
